# Toward a High-Resolution Neuroimaging Biomarker for Mild Traumatic Brain Injury: From Bench to Bedside

**DOI:** 10.3389/fneur.2015.00148

**Published:** 2015-07-06

**Authors:** Manoj Kumar Jaiswal

**Affiliations:** ^1^Center for Neuroscience and Regenerative Medicine, Bethesda, MD, USA; ^2^Department of Anatomy, Physiology and Genetics, School of Medicine, Uniformed Services University of the Health Sciences, Bethesda, MD, USA

**Keywords:** traumatic brain injury, neuroimaging, biomarker, magnetic resonance imaging, concussions, blast injury, diffusion tensor imaging, voxel-based morphometry

## Introduction

**Figure 1:**
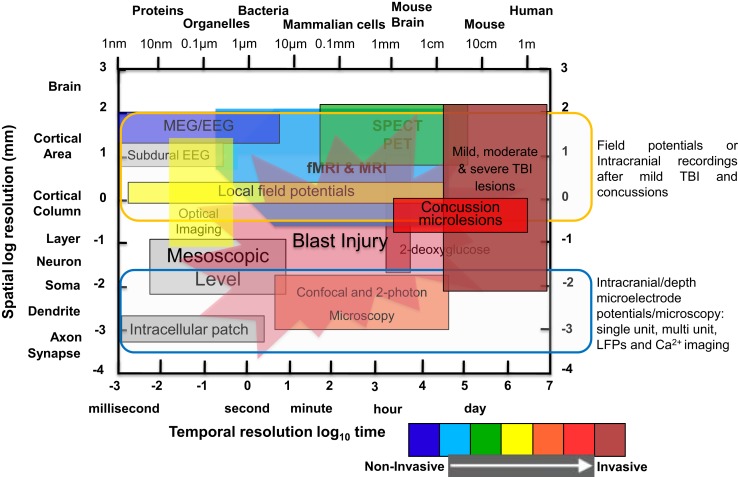
**Comparison of spatial and temporal resolution and penetration depth of different neuroimaging techniques used in clinical and laboratory setting to investigate mild TBI, moderate/severe TBI, and blast injury**. Illustration of functional neuroimaging and neurophysiological techniques showing comparison of spatiotemporal resolution and penetration depth of neurometabolic optical techniques. The *x*-axis (time in seconds to day or size of object/animal/patients) and the *y*-axis (distance in millimeters) are scaled logarithmically. Penetration depths are color-coded from non-invasive to invasive, ranging from blue to red color [modified from Ref. (4–8)].

Mild traumatic brain injury (mTBI) continues to be a major public health issue among our active military, veterans, and society at large. The Centers for Disease Control and Prevention (CDC) estimates that ~1.5 million people annually survive a TBI and ~230,000 require in-patient treatment. To fully gauge the efficacy of emerging therapeutic drug candidates for the treatment of mTBI, reliable biomarkers have to be identified for early detection of brain injury and subsequent prediction of the outcome. Guidelines about the use of high-resolution neuroimaging techniques in the treatment and management of mTBI recognized the substantial contribution of magnetic resonance imaging (MRI), positron emission tomography (PET), computed tomography (CT), and 2-photon imaging for their sensitivity in visualizing white matter (WM) tracts and sensorimotor circuits (1–3). MRI imaging, with its increased sensitivity to visualize WM tracts and sensorimotor circuits, the cerebellum, and extra-motor pathology and pathways, thus, is a favored approach in the search for biomarkers. The power of cutting edge imaging methodologies, in combination with collaboration and data sharing among numerous centers, is recognized in neurodegenerative research. Such a multicenter, collaborative approach for the study of neuroimaging biomarkers for mTBI has the prospect to generate data sets large enough to judge the feasibility of MRI as an outcome measure for different treatment strategies. A comparison of spatiotemporal resolution and penetration depth of various neuroimaging methods compared to MRI is shown in Figure 1.

The main goal is to formulate guidelines on MRI imaging protocols for studies of mTBI focused on four broader research goals.

1. Optimize matching of patients to available therapies for personalized mTBI treatments.
2. Develop effective mTBI patients outcome measures for drug treatments.
3. Design one-to-one personalized novel therapeutic strategies on individual basis.
4. Identify promising intervention strategies and execute it for armed forces patients and civilians.

### Diffusion tensor imaging

Diffusion tensor imaging (DTI) is a MRI method that is susceptible to the direction of water movement. It has the most sensitive and predictive MRI metric in TBI and can be used to detect pathology within neuronal WM tracts (1, 9). In mTBI, this technique effectively differentiates patients with mTBI from control patients, regardless of severity and time frame following injury, and reveals neuropathological patterns that were previously only observed in histological studies post-mortem. Specific to mTBI, the fractional anisotropy component of DTI has yielded the most reliable results. In a study examining 30 war veterans with a history of mTBI, with a subgroup of 13 showing impaired neuropsychological performance, evaluated as executive function (EF) measure, DTI detected WM differences that correlated with reduced EF performance (10). Damage to tracts of WM causes freer movement of water molecules in these areas, resulting in a decrease in the fractional anisotropy value which can function as a measure for the extent of injury. DTI biomarker findings provide novel information about brain-behavior relations that could never be gleaned from just the neuropsychological data, since group averaging neuropsychological test findings may obscure those with mTBI. However, DTI has so far proved inconsistent in the diagnosis of soldiers with blast-related mTBI and there is no consensus on the ideal method because of the small numbers of patients studied. Human study conducted on 63 US military personnel within 90 days of blast-related mTBI, all had normal CT, but 18 patients had abnormalities on DTI consistent with diffuse axonal injury (11). A new mode of MRI, called super-resolution track diffusion imaging, produces high-resolution fiber tracking and might have application in the detection of subtle abnormalities after mTBI (12). While showing great potential as a clinical biomarker or for predicting mTBI outcomes, further long-term studies are needed to understand the true value of DTI in this field.

### Voxel based morphometry

Voxel based morphometry (VBM) is a high-resolution neuroimaging analysis technique that detects differences in brain anatomy between an averaged brain image template and high-resolution three-dimensional T1-weighted MRI scans of study subjects with an automated analysis of gray or WM volume. For Alzheimer’s and Huntington’s disease, VBM analysis of MRI images is the predominant method to monitor progression of degeneration. In mild and severe TBI, VBM has been consistently sensitive to somatosensory and cerebral changes, demonstrating the pathological similarities of mTBI with dementia. However, lack of longitudinal MRI studies and contradictory results of sensory atrophy put the question marks on sensitivity of VBM to disease progression in mTBI (1). In a meta-analysis of 39 studies of hippocampal volumes in adults, Woon and colleagues described reduced hippocampal volume after psychological trauma, which was worse when PTSD was present after blast-related injury (13).

### Functional MRI

Functional MRI (fMRI) utilizes benefit of susceptibility variations among oxygenated and deoxygenated blood and measures the rate of blood flow indirectly. Blood oxygenation level dependent (BOLD) signal with a sensitivity of few seconds correlates with alterations in neuronal activity and provides confirmation for global changes in cortical activity as a reliable feature of mTBI, concussion, and blast-related TBI pathology. Blood flow aberrations, particularly in the frontal lobes, highlighted after the studies of working memory in mTBI and sTBI. To study working memory in mTBI patients with normal structural scans, 12 mTBI patients [Glasgow coma score (GCS) of 13–15, duration of loss of consciousness (LOC) ∼30 min] were tested during 6–35 days post mTBI. In comparison to control subjects, mTBI patients had difficulty in concentrating and doing routine job but there was no difference in anxiety levels or depression. While mTBI patients had poor reaction time and attention deficits (neuropsychological measures), mTBI patients perform equally well compared to control subjects for other measures e.g., EFs and memory. mTBI patients were asked to perform the “n-back” task related to working memory, where mTBI patients memory were tested through the presentation of a chain of letters. mTBI patients were asked to distinguish whether a letter presented represented an object letter presented visually a minute before, or whether the letter heard matched the letter seen two letters preceding during the 1-back and 2-back condition, respectively. Patients and control subjects show activated bilateral frontal and parietal regions, but the pattern is different in control subjects where there is increase in activation from 0-back to 1-back compared to mTBI patients, showing increases in activation from 1-back to 2-back (14–16). Four football players having concussion (no LOC but transient confusion) and exposed to mTBI, fMRI study shows no reliable change in test scores for sensory coordination and working memory within 1-week post injury (17). Compared to control subjects, concussed players 1-week post mTBI showed increased activation in motor and premotor cortex, superior and inferior parietal regions, and bilateral cerebellar regions. Although these studies meet some of the criteria e.g., sensitivity, validity, and functional correlates, further studies are needed to provide answer for difficulty in controlling mood and susceptibility. In spite of these issues, fMRI has offered a number of promising results to date in mTBI patients.

Application of resting-state functional MRI (rs-fMRI) to patients with mTBI suggests that reduced inter-hemispheric hemorrhage and functional connectivity between motor and sensory cortices is a feature of early symptoms and might be used as a biomarker for detection of mTBI disease (18). Use of rs-fMRI for mTBI patients suggests that reduced inter-hemispheric hemorrhage and decreases in functional connectivity between motor and sensory cortices are a characteristic features of mTBI and can be used as an early biomarker for diagnosis.

### Magnetic resonance spectroscopy

Magnetic resonance spectroscopy (MRS) technique is used to evaluate the metabolic condition of the brain non-invasively after mTBI using positively charged proton-based cerebral metabolites presented as a ratio with creatine or choline. While DTI examines anatomy after injury, MRS measures brain metabolism, in particular, the relative amounts of specific metabolites in brain tissue shown to be perturbed following mTBI, such as *N*-acetylaspartate (NAA, a marker of network integrity), ATP, lactate, choline, and glutamate in patients after mTBI, concussion, and blast injury (19, 20). MRS is also useful in observing cellular reaction to drugs interventions and detecting alterations in high-energy metabolites such as phosphates dysregulated after mTBI. MRS with strength 3T and above is able to separate neurochemical’s peaks, e.g., glutamate and GABA, and consider as a prominent biomarker for pathogenesis of mTBI. There are several barriers that need to be conquered to do high-quality MRS imaging, e.g., lack of acquisition standardization (single-voxel vs. multivoxel sampling), interpretation of individual studies, and generalization to clinical practice, undersized mTBI patients study, subject selection factor, etc.

## Limitations, Conclusion, and Future Directions

The intent of this brief prospective was an update on high-resolution neuroimaging biomarkers for mTBI that could provide the basis for a sensitive, objective metric to enhance detection of mTBI sequelae. This could form the basis for improved and complementary research protocols for studies of brain injury and concussion, and aid the design for investigations of neuropsychological outcome. While specifics like severity or progression are important for future treatments, in my view, an accurate diagnosis of mTBI is the first most important criteria. By obtaining pre-injury exposure studies, and by more accurately identifying pre-mortem and studying disease progression, we can establish further insight into the effects of mTBI and improved therapeutic approaches for cure. One of the key potential uses for neuroimaging data is the prediction of recovery after brain injury. The application of novel MRI methods, e.g., DTI, VBM, MRS, and fMRI, has been extensively reported in diagnosis of stroke pathology, but so far there is limited use of this technology in patients having brain trauma and concussions. From a clinical perspective, before neuroimaging methods apply, demographic information (age/sex), date of injury, ethnic origin, family history, date of symptoms onset, date of diagnosis by experts, and other variables (e.g., merging structural and functional imaging studies) are very important for valid analysis of the imaging data.

While this prospective highlights the merit of potential neuroimaging biomarkers of mTBI, neuroimaging remains an expensive study method and cost of neuroimaging is a legitimate concern for low income patients. Another legitimate concern is that the brain may adapt to mild injury, and therefore, positive imaging results would possibly reflect an abnormality to which the brain has already adapted and recircuited without apparent ill effect. A recent study demonstrated in their prospective cohort of those who sustain mTBI, a prior concussion distinctly and significantly prolonged symptoms and put forward the concept of “recovered” mTBI even in the presence of a lesion (21). This is a serious issue for clinicians and researchers to contemplate before they would conclude that “no injury” had occurred based on neuropsychological and behavioral findings alone, and that there were “no untoward” effects of mTBI. Restoration of neural function is likely the norm following mTBI, but contemporary neuroimaging methods might identify residual indicators of neuropathology in subsets of individuals with mTBI.

Nevertheless, neuroimaging provides biomarkers of underlying structural and physiological abnormalities in TBI, concussion, and blast-related injury, and these pathological changes occur in regions and within neural systems that plausibly give rise to the common types of neurobehavioral and neurocognitive sequelae associated with mTBI that need to be incorporated into neuropsychological outcome studies. The major focus of neuroimaging studies should be to identify pathology potentially related to residual impairments in cognitive and/or behavioral functioning post-mTBI. The use of various MRI techniques in clinical settings and connecting structure and function by combining rsfMRI with DTI and VBM might advance sensitivity and specificity to diagnose mTBI and concussed patients as demonstrated earlier where mTBI patients in which the combination of cortical, GM/WM, and hippocampal DTI, VBM, fMRI, and MRS resulted in 75% for all four indices and 90% for first two indices (1–3, 22). Accord is slowly arriving about essential and desirable protocols for MRI for forthcoming studies of mTBI with high aims for collaborative multi-center longitudinal studies. The use of neuroimaging based biomarkers for mTBI has a broad range of potential use that includes whole brain system biology, brain area specific, and connectomics study, which might lead to the discovery of multiple therapeutics and efficient clinical trials with the hope of translating findings into a better future for patients. Furthermore, use of neuroimaging as a diagnostic (biomarker) tools for detection of functional, morphometric, and chemical changes might leads to the evaluation of therapeutics measures, e.g., drugs effects measures, behavioral correlates, etc. (23–25).

## Conflict of Interest Statement

The author declares that the research was conducted in the absence of any competing commercial or financial conflict of interest. The views expressed in this article are those of the author and do not reflect the official policy or position of the Department of Defense, and the United States Government.
